# Three-Dimensional Evaluation of Maxillary Sinus Changes in Growing Subjects: A Retrospective Cross-Sectional Study

**DOI:** 10.3390/ma13041007

**Published:** 2020-02-24

**Authors:** Cinzia Maspero, Marco Farronato, Francesca Bellincioni, Alessandro Annibale, Jacopo Machetti, Andrea Abate, Davide Cavagnetto

**Affiliations:** 1 Department of Biomedical, Surgical and Dental Sciences, School of Dentistry, University of Milan, 20100 Milan, Italy; marco.farronato@unimi.it (M.F.); francescabellincioni@hotmail.com (F.B.); ale.annibale@gmail.com (A.A.); jaco.machetti@gmail.com (J.M.); andreabate93@gmail.com (A.A.); davide.cavagnetto@gmail.com (D.C.); 2 Fondazione IRCCS Cà Granda, Ospedale Maggiore Policlinico, 20100 Milan, Italy

**Keywords:** cone-beam computed tomography, maxillary sinus, dental radiography, dental informatics, digital dentistry, growth and development

## Abstract

This study aims to evaluate changes of maxillary sinuses in growing subjects. Cone Beam Computed Tomography (CBCT) scans of 146 patients were divided according to gender and age (6–8, 9–11, 12–14 years old). Left, right and total maxillary sinus volume (MSV-R, MSV-L, MSV-Tot) and surface (MSS-R, MSS-L, MSS-Tot), left and right linear maximum width (LMW-L, LMW-R), depth (LMD-R, LMD-L) and height (LMH-R, LMH-R) were calculated using Mimics Research 22. Kruskal–Wallis Test and showed a statistically significant increase in both genders for all variables. Pairwise comparisons in females are always statistically significant in: LMH-R, LMH-R, MSS-Tot, MSV-Tot. All other variables showed a statistical significant increase between 9–11 and 12–14, and between 6–8 and 12–14 age groups, apart from LMSW-R, LMSW-L, LMSD-R, LMSD-L between 6–8 and 12–14 age groups. Pairwise comparisons in males are always and only statistically significant between 9–11 and 12–14, and between 6–8 and 12–14 groups. Symmetrical measurements (right and left) evaluated using Wilcoxon test retrieved no statistical significant difference. Comparisons between measurements on male and female subjects using Mann–Whitney test showed a statistical significant difference in 6–8 years group in MSV-R, MSV-L and MSV-Tot, and in 12–14 age group in MSV-R, MSV-L, MSV-Tot, MSS-r, MSS-l, MSS-Tot, MSW-R, MSW-L, MSD-R, MSD-L. Intraclass Correlation Coefficient (ICC) assessing inter-operator and intra-operator concordance retrieved excellent results for all variables. It appears that maxillary sinus growth resembles the differential peak of growth in male and female subjects. Sinuses starts to develop early in female subjects. However, in the first and last age group female sinuses are statistically significantly smaller compared to male ones. In male subjects, sinus growth occurs mainly between the second and third age group whilst in female subjects it starts between the first and second age group and continues between the second and the last. Sinus has a vertical development during the peak of growth, which is the main reason for its increase in volume.

## 1. Introduction

Maxillary sinuses are the largest amongst paranasal sinuses [1,2]. They are a pair of bony chambers located inside each maxillary bone. The knowledge of maxillary sinus anatomy is important in forensic medicine and in several dental and maxillofacial procedures.

Their growth starts during the 3rd month of fetal development as an evagination of the epithelium of the lateral wall of the nasal fossa [3]. Postnatal growth of maxillary sinus according to present literature happens mainly during the first three years of life and between 7 and 12 years of age [4,5]. They reach their adult size between 12 and 15 years of age [6].

Maresh first reported observations and measurements of maxillary sinus changes during adolescence assessed on [7]. He discussed growth in size of the maxillary sinus and its great variability depending on subject [7]. Koymen et al. stated that size varies from one individual to another and facial morphology seems to reflect sinus dimensions [8].

Several studies present in literature regarding the development and size of the maxillary sinuses, have shown different results. One of the possible reasons is that maxillary sinus volume (MSV) have been evaluated in various methods in literature such as two dimensional radiographs, stereology, injection of various substance into the maxillary sinus and use of the ellipsoid formula [9,10,11].

Recent decades have seen the development of different imaging modalities that have revolutionized dental and medical diagnosis. Cone Beam Computed Tomography (CBCT) was introduced in 1998 and it has become more and more frequently used for clinical and research purposes in dentistry [12]. It represented a real revolution as it could provide accurate and distortion free images of the craniofacial bones and a lower absorbed radiation dose compared with multi slice computed tomography (MSCT) [13,14,15].

CBCT scans elaborated with dedicated 3D softwares allow to evaluate all craniofacial structures three dimensionally to precisely perform morphometric measurements.

Growth of maxillary sinuses in young patients is scarcely investigated in literature and it is often studied using bidimensional radiographs as ortopathomography and cephalometric radiographs. As the maxillary sinus is a three-dimensional (3D) structure, analyses based only on conventional two-dimensional (2D) radiographs alone can result in limited information and erroneous conclusions. A more accurate approach is to analyze CBCT that provides 3D multi-planar images and information on maxillofacial regions especially in maxillary sinuses.

In the last few years some studies have evaluated the volume of the maxillary sinuses and upper airway for surgical and medical purposes [10,16,17,18]. Recent studies evaluate the changes of maxillary sinus volume according to age and gender in adult patients [19]. To the best of our knowledge no studies in literature have evaluated changes in maxillary sinus in growing patients.

The purpose of this study was to evaluate the maxillary sinus volume (MSV), maxillary sinus surface (MSS) and linear maximum width (LMW), height (LMD) and depth (LMH) of the maxillary sinus in growing patients and to compare them according to their sex and ages, in order to highlight any morphological changes due to patient growth.

## 2. Materials and Methods

The study, presented herein, was approved by the competent IRB inside the research project of the year 2018 O.U. N. 420/425 of Fondazione IRCCS Cà Granda Ospedale Maggiore Policlinico, Milano, Italy. All patients’ parents gave written informed consent to all the procedures performed. All procedures were carried out in accordance with the ethical standards and with the Helsinki Declaration of 1975 and revise in 2000.

### 2.1. Sample Selection and Inclusion Criteria

This retrospective study involved the use of CBCT scans of patients referred for various reasons to the Department of Radiology of Fondazione IRCCS Cà Granda Ospedale Maggiore Policlinico between 2009 and 2018. Maxillofacial CBCT scans including the maxillary sinuses were acquired for the following reasons: orthognathic surgery, third molar surgical evaluation, orthodontic evaluation of unerupted teeth, cysts of the maxillary sinus, odontogenic or non-odontogenic cysts which had not affected the maxillary sinus.

Inclusion criteria were:

- Growing patients,
- Caucasian patients.

Exclusion criteria were:

- pathological conditions affecting the maxillary sinuses (fracture, inflammation, residual root fragments, extrusion of endodontic filling materials),
- missing teeth in maxillary posterior regions,
- history of orthodontic treatment,
- metabolic diseases affecting bone tissue,
- skeletal asymmetry,
- congenital disorders,
- craniofacial syndromes.

Records of 1600 patients were reviewed. CBCT scans of 146 Caucasian patients (74 males and 72 females) who met the study criteria were included in the study. This sample was divided according to their age into three age groups: group one 6–8 years (28 males; 27 females), group two 9–11 (23 males; 22 females), group three 12–14 (21 males; 25 females). Male and female subjects were analyzed separately and then each age group was compared between genders

Right and left maxillary sinuses of each patient were calculated. The following parameters were evaluated: left, right and total maxillary sinus volume (MSV-R, MSV-L, MSV-Tot) and surface (MSS-R, MSS-L, MSS-Tot), left and right linear maximum width (LMW-L, LMW-R) depth (LMD-R, LMD-L) and height (LMH-R, LMH-R) were calculated using Mimics Research 22

### 2.2. CBCT Examination and Data Processing

All the images were obtained for all patients using the same I-CAT FLX (Imaging Sciences International, Hatfield, PA, USA) with the same exposure parameters. The scanning parameters were configured as follow: 360° rotation, 300 frames, 120 kV[p], 5 mA, 3.7 s, voxel size 0.4 mm, field of view (FOV) 16 mm × 8 mm/16 mm × 11 mm, to minimize radiation exposure.

All images were saved in the Digital Imaging and Communications in Medicine (DICOM 3) format. DICOM files were elaborated by an expert radiologist. These data were then imported into the Mimics Research^TM^ software version 21.0 (NV, Technologielaan 15, 3001 Leuven, Belgium) where all the measurements were performed.

**Figure 1:**
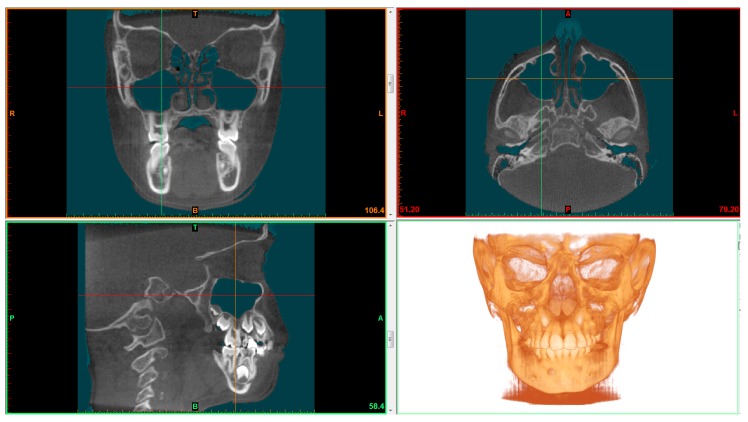
Sagittal, coronal and axial view after thresholding.

**Figure 2:**
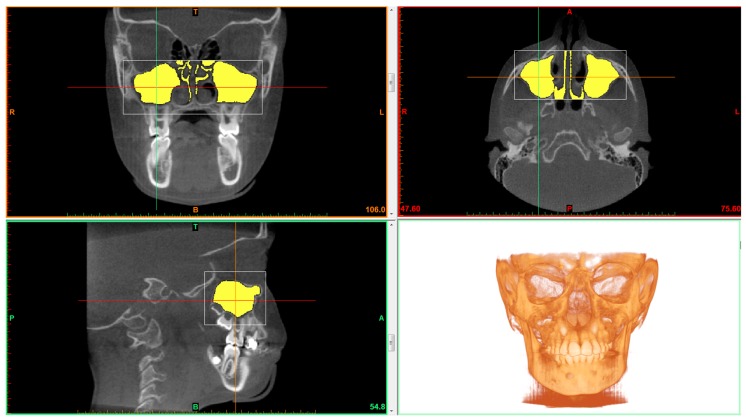
Sagittal, coronal and axial view of the maxillary sinus. The isolated maxillary sinuses are clearly visible.

Thresholding was applied for maxillary sinus volume calculation. Thresholding limits were −1024 HU (minimum) and −526 HU (maximum) (Figure 1) [17]. Maxillary sinus was cropped using the software’s tool called “edit masks” along the borders described by Motro et al. [20]: the bone structure and the narrowest space of the ostium between the infundibulum and processus uncinatus. The connection with the outer air was then cropped slice by slice using the segmentation tools. The “region growing” tool was used to split the segmentation created by thresholding into several objects and to remove floating pixels. (Figure 2) Maxillary sinus volumes were calculated using the software’s “calculate 3D” tool [19].

**Figure 3:**
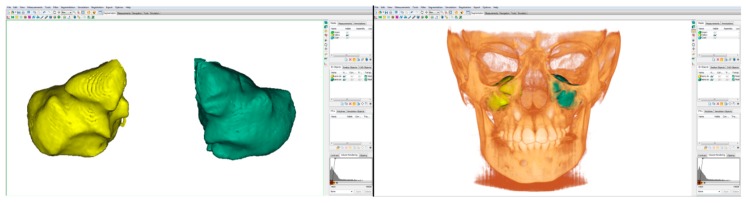
Three-dimensional image of the right and left maxillary sinuses.

After segmentation, the three-dimensional volumetric structure of the maxillary sinus volume (right and left) of each patient was calculated separately. Flood-fill and smoothing operations were applied to the airway mask, in order to calculate the total volume with no regard of porosity in the MSV (Figure 3). The software Mimics^TM^ then automatically calculated volume, surface of the selected structures. Linear maximum width (LMW), height (LMH) and depth (LMD) were calculated by placing points in the areas of maximum anterior and posterior depth, maximum upper and lower height and maximum width to the right and left for each maxillary sinus. The Mimics Research^TM^ software version 21.0 (NV, Technologielaan 15, 3001 Leuven, Belgium) automatically calculate the distance between these points in order to return the maximum depth, width and height.

### 2.3. Statistical Analysis

The software SPSS (Statistical Package for Social Sciences version 25.0, Chicago, IL, USA) was used for the statistical analysis. Dimensional variations between right and left maxillary sinus volume (MSV), maxillary sinus surface (MSS), linear maximum width (LMW), height (LMH) and depth (LMD) of the maxillary sinus in the three age groups and between male and female were analyzed.

The Kolmogorov–Smirnov test was used to assess whether the data was normally distributed. The statistical distribution of the quantitative measures was found not to be Gaussian. Each measurement has been reported as mean and standard deviation.

Test U of Mann–Whitney and Kruskal–Wallis test for not normally distributed data were used for statistical analysis. Test U of Mann-Whitney was used to compare the difference between males and females and Wilcoxon signed rank test for right and left side in each group for the following parameters: maxillary sinus volume (MSV), maxillary sinus surface and maximum width (LMW), height (LMH) and depth (LMD) of the maxillary sinus. Kruskal–Wallis test was used to detect differences in the three groups for male and female analyzing maxillary sinus volume, maxillary sinus surface and linear maximum width (LMW), height (LMH) and depth (LMD) of the maxillary sinus. The post hoc Wilcoxon test with Bonferroni’s correction was used for within-group comparisons. *P* value < 0.05 was considered statistically significant.

### 2.4. Method Error

All the CBCT scans were taken and analyzed by an expert radiologist. Measurements were obtained by a single expert orthodontist specialized in 3D radiologic imaging who performed all the measurements. After two weeks, 50 CBCT randomly selected by the sample were examined by a different operator and then recalculated by the first one checking MSV, MSS, maximum width (LMW), height (LMH) and depth (LMD) of the maxillary sinuses in order to assess intraobserver and interobserver reliability.

## 3. Results

Descriptive statistics and statistical comparisons of MSV, MSS, linear maximum width (LMW), height (LMH) and depth (LMD) measurements of the maxillary sinus between right and left side and sex in each group are shown in Table 1.

### 3.1. Comparisons Whithin Groups between Genders

#### 3.1.1. Maxillary Sinus Volume (MSV)

MSV results showed statistically significant difference when comparing the males and females in group 1 (age 6–8) and group 3 (age 12–14). No statistically significant difference has been noticed between right and left side of the same groups (Table 1).

No statistically significant difference was found in group 2 (age 9–11) for MSV between right and left side in male and female subjects.

#### 3.1.2. Maxillary Sinus Surface (MSS)

No statistically significant difference was found in group 1 (age 6–8) and group 2 (age 9–11) for MSS between right and left side and sex.

MSS in group 3 (age 12–14) showed statistically significant difference between male and female subjects. No statistically significant difference was found comparing right and left side (Table 1).

#### 3.1.3. Linear Maximum Width (LMW)

Results showed no statistically significant difference in group 1 (age 6–8) and group 2 (age 9–11) for Maximum width (LMW) between right and left side and sex. Statistically significant differences were found in group 3 (age 12–14) comparing male and female (Table 1).

#### 3.1.4. Linear Maximum Depth (LMD)

No statistically significant difference was noticed in group 1 (age 6–8) and group 2 (age 9–11) for maximum depth (LMD) between right and left side and sex. Maximum depth (LMD) in group 3 (age 12–14) showed statistically significant difference between male and female and no statistically significant difference was found comparing right and left side (Table 1).

#### 3.1.5. Linear Maximum Height (LMH)

Results showed no statistically significant difference in group 1 (age 6–8), group 2 (age 9–11) and group 3 (age 12–14) for Maximum height (LMH) comparing right and left side and sex (Table 1).

### 3.2. Comparisons whithin Gender between Age Groups

Statistical comparisons of MSV, MSS, Maximum width (LMW), height (LMH) and depth (LMD) measurements of the maxillary sinus between the three different groups divided for sex are shown in Table 2 and Table 3. Kruskal–Wallis test demonstrated a statistically significant difference for all the variables considered in both genders. (Table 2 and Table 3)

In female subjects Wilcoxon test highlighted statistically significant difference between groups 1 (age 6–8) and 2 (age 9–11) for the following parameters: MSV Tot, MSS Tot, Maximum height right and left (Table 2).

**Figure 4:**
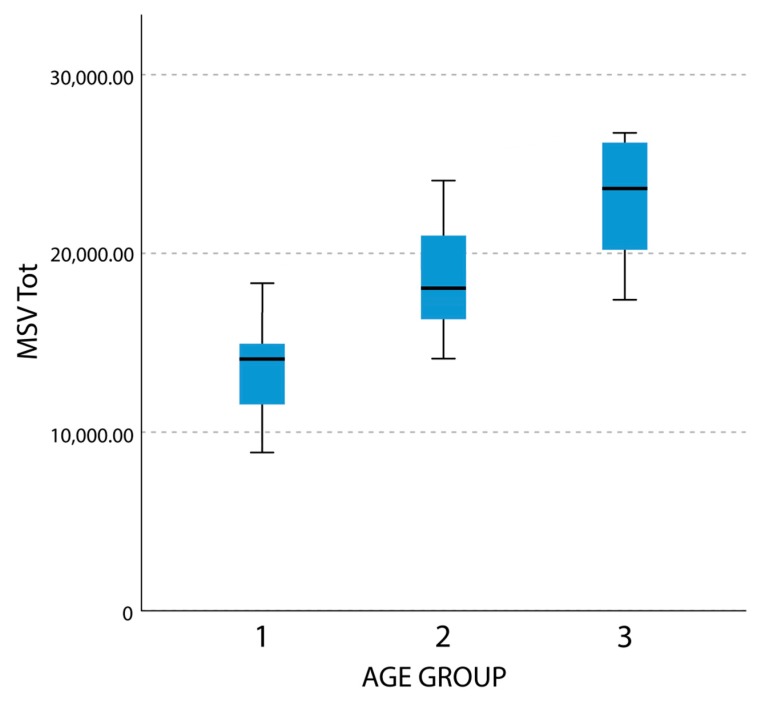
Boxplot representing total maxillary sinus volume (MSV Tot) in female subjects.

Comparisons between groups 2 (age 9–11) and 3 (age 12–14) showed statistically significant differences for the all the parameters except for maximum width (LMW) and maximum height (LMH) in both sides. Differences between groups 1 and 3 noticed a statistically significant difference for all the parameters. Results for female subjects are summarized in Table 2. Figure 4 reports the self-explanatory example of MSV Tot boxplot in female subjects.

**Figure 5:**
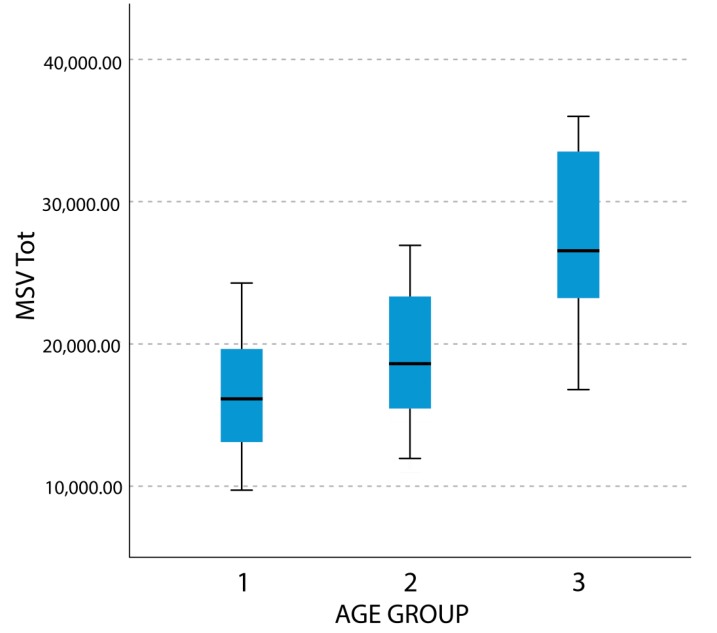
Boxplot representing total maxillary sinus volume (MSV Tot) in male subjects.

In male subjects, Wilcoxon test noticed no statistically significant difference for all the variables between groups 1 and 2 contrary to the comparison between group 3 with groups 1 and 2 that highlighted a statistically significant difference for all the variables. Results for the male group are summarized in Table 3. Figure 5 reports the self-explanatory example of MSV Tot boxplot in male subjects. By comparing the visual difference between Figure 4 and Figure 5, the reader may better understand the difference in timing of development of maxillary sinuses between male and female subjects.

The intraobserver and interobserver reliability showed high agreement for all the variables evaluated, average (± SD, range) intraobserver and interobserver ICC were respectively: 0.978 (±0.065, 0.967–0.989) and 0.968 (±0.012, 0.944–0.983) (Table 4).

## 4. Discussion

Different studies have investigated maxillary sinus volume and their volumetric changes induced by several conditions such as orthodontic treatment, septum deviation, and sinus pathologies. Authors also investigated the relationship between maxillary sinus dimension with race, sex and age [21,22,23].

Koppe et al. in 2006, when comparing the maxillary sinus volume and facial skeleton of adult skull with non-treated bilateral cleft with adult normal skulls without pathologies showed that bigger skulls tended to possess larger maxillary sinuses [24], this is also confirmed in the studies of Barbosa [25] in 2014 and Erdur [26] in 2015 where they noticed that the volume of the maxillary sinus was negatively affected in patients with cleft palate and lip.

Ariji et al. reported that the MSV increased in subjects up to 20 years of age and then decreased. In addition, they found no significant difference between the right and left MSVs or between sexes [27]. Our findings are in agreement with that reported by Ariji since no significant difference between the right and left MSVs was noticed in each group and for all the variables evaluated (Table 1).

Kurita et al. [28] in 1988 and Lee DH [29] in 2005, reported differences in the size of the maxillary sinus depending on sex, whereas Graney DO [30] in 1993 reported no statistically significant differences between genders.

Oktay [2] in 1992 measured the maxillary sinus areas on panoramic films in patients with ideal occlusions and with malocclusions and he found that malocclusions and sex factors have no effect on the size of the maxillary sinuses, however gender is significant only in female subjects with Angle Class II malocclusions which have larger maxillary sinuses. Our study conducted on CBCT of young patients yielded different results compared with Graney and Oktay. In fact, statistically significant difference was evaluated for MSV between male and female in the groups 1 (age 6–8) and 3 (age 12–14). Maxillary sinus surface, maximum width (LMW) and maximum depth (LMD) highlighted a statistically significant difference between sexes only in the group 3. These results are probably due to the fact that male patients in group 3 were during the pubertal peak of growth and the continuous development of the cranio-facial structures could lead to an increase in the maxillary sinus. Furthermore, this difference is thought to be due to sexual dimorphism.

In 2010 Endo T et al. [4] measured the maxillary sinus areas in different malocclusion classes on lateral cephalometric headfilm. They found that patients 12 to 16 years old with large cranial bases and nasomaxillary complexes tend to have larger maxillary sinuses.

The subjects involved in our study had not undergone any orthodontic extractive treatments and they had no other dental pathological conditions. As reported in literature orthodontic treatment seems to affect the maxillary sinus volume and size. In extraction cases moving teeth through sinus area is important factor for new bone apposition [31,32]. Oz et al. [33] also found that maxillary sinus volume was affected by impacted teeth and another pathologic situation.

Growth and development of the maxillary sinus occurs in the mid-face, but no study has examined the changes in size and volume of the maxillary sinus during growth. Jun et al. [29] reported that maxillary sinuses continue expanding until the second decade of life in females and until the third one in males that afterwards they slightly decrease. Jun et al. also stated that MSV is significantly bigger in males than in females during the developmental period.

However, our results show that the differences in maxillary sinus dimensions (MSV, MSS, width, depth, height) between males and females are statistically significant mainly in group 3. In fact, sinus development appears to approximately follow pubertal peak of growth. Concerning the pubertal growth spurt, sinus development in males occurs later than in females. The difference in maxillary sinus volumes between genders in group 1 appears reduced in group 2, albeit present, and, finally, more evident in group 3 (see Table 1). Comparison of sinus dimensions within the three groups in female subjects demonstrated that maxillary sinuses start growing early compared to males as they already present a statistically significant difference between group 1 and group 2 for the following parameters: MSV Tot, MSS Tot and Maximum height R and L. Sinus growth continues in female subjects between group 2 and 3. The following parameters showed a statistically significant difference: MSV R, MSV L, MSV Tot, MSS R, MSS L, MSS Tot and maximum height R and L. Between group 1 and group 2, all the variables presented a statistically significant difference.

In male subjects, sinus development appears to be different. Between group 1 and group 2 no measurement presented any statistically significant difference. Between group 2 and group 3 and between group 1 and group 3 all measurements presented a statistically significant increase. Mean male maxillary sinuses are bigger in the prepubertal period, and they start growing later compared to female ones but at the end of pubertal peak of growth appear statistically bigger.

Height appears as the main factor for maxillary sinus increase in volume and surface over time both in females and in males. Maxillary sinus height is the parameter that mostly changes throughout growth. Intraclass Correlation Coefficients showed excellent intra-operator and inter-operator agreement (see Table 4) in the evaluation of maxillary sinus dimensions using the presented method.

Improving knowledge on the development of the maxillary sinuses is important in many dental disciplines. The floor of the maxillary sinuses is formed by the palatine process of the maxilla and its contiguity with the upper posterior teeth continues to change throughout life [2]. Several studies investigated the side effects that somehow involve the maxillary sinuses and that may arise following common dental procedures [34,35]. They usually focus on side effects following endodontic treatment [36] or implant placement [34,37] and their possible prevention and/or treatment. A deeper knowledge of its three-dimensional changes could be useful even in orthodontics [2]. In fact, orthodontic treatments involving sliding mechanics of posterior maxillary teeth, especially if one or more of them are missing, should be performed after an accurate evaluation of the relationships between the apexes of the aforementioned teeth and the cortical floor of the maxillary sinuses that could represent an obstacle to their correct movement [38,39]. Tooth movement in cortical bone is indeed considered an anatomical limitation in orthodontics because of its slower turnover and higher density [40,41,42]. In these cases, teeth seem to easily loose anchorage and get tipped, potentially resulting in root resorption [43]. More knowledge on the timing of three dimensional maxillary sinus development could be helpful in treatment planning especially because of its prevalent vertical development. However, these considerations, albeit worthy of further examination, are beyond the aim and possibility of the present study that mainly focuses on morphological changes of the sinuses.

The limitations of this study include a relatively small sample and the impossibility of having images of the same patients over time because of the invasiveness of the CBCT exam. The continuous development of the magnetic resonance imaging (MRI) technology and of the techniques for its digital elaboration will hopefully overcome the burden of radiation dose connected to CBCT imaging [44,45,46]. As a result of the increasing application of MRI in dental imaging, which is already happening [44,45,47], researchers will be able to better understand the development of facial structures through aging. The evaluation of maxillary sinuses in MRI could lead to a better comprehension of the modifications that occur inside the maxillary sinuses not only through aging but also in response to pathologies or to the movement of maxillary posterior teeth thereby improving the comprehension of the pathophysiological mechanisms that rule bone metabolism in the maxillary sinuses without radiation exposure.

## 5. Conclusions

There is no significant difference in age group 1, age group 2 and age group 3 between right and left maxillary sinus dimensions in both genders.

The maxillary sinuses of male subjects showed a statistically significant increase mainly between group 2 and group 3, which corresponds to the male pubertal peak of growth. The maxillary sinuses of female subjects started to develop early showing a less steeper increase. Indeed, a statistical significant increase was noted between group 1 and group 2 and between group 2 and group 3. Therefore, the development of the sinuses of female subjects occurred early compared to male as their peak of growth. The development of the maxillary sinuses in both sexes seems to overlap with the peak of growth, with a development that begins in females between 9 and 11 years old and in males between 12 and 14 years old.

## Figures and Tables

**Table 1:** Descriptive statistics for maxillary sinus parameters; Wilcoxon tests between right and left side and Mann–Whitney test to compare Males vs. Females.

Measurements		Male			Female			Comparison Male vs. Female
		Mean; ± SD	Median; (1–3 quartile)	R vs. L *p* Value	Mean ± SD	Median; (1–3 quartile)	R vs. L *p* Value	*p* Value
MSV-R	1	8493.15 ± 2475.17	8054.94 (6758.08; 9987.84)	0.921	7165.59 ± 1852.45	6872.39 (6266.72; 7861.48)	0.341	0.027 *
MSV-L	1	8462.65 ± 2782.68	8043.21 (6232.69; 9055.70)		6907.22 ± 2051.71	7008.02 (6199.91; 8345.58)		0.041 *
MSV-Tot ^a^	1	16955.79 ± 5013.80	16131.32 (12765.36; 19647.39)	/	13998.74 ± 3687.55	14474.08 (11783.02; 15378.44)	/	0.035 *
MSV-R	2	9192.22 ± 2657.30	8912.55 (6971.69; 12251.59)	0.982	8616.21 ± 2211.75	8291.05 (7246.57; 9792.60)	0.614	0.525
MSV-L	2	9187.38 ± 2359.84	8781.82 (7234.86; 10888.86)		8739.56 ± 2104.15	8842.62 (7265.33; 10333.56)		0.683
MSV-Tot ^a^	2	18379.59 ± 4924.78	17666.02 (14194.21; 22615.68)	/	17033.04 ± 3704.71	16233.36 (14853.99; 19878.85)	/	0.414
MSV R	3	13760.25 ± 2987.21	13271.63 (11189.04; 16610.20)	0.914	12072.57 ± 1456.78	11802.14(11263.35; 13552.32)	0.600	0.025 *
MSV-L	3	13716.68 ± 2915.70	13672.61 (10852.76; 16749.64)		11935.12 ± 2211.45	12913.72 (10024.76; 13737.23)		0.02 *
MSV-Tot ^a^	3	27476.93 ± 5613.75	26543.26(23051.89; 33593.30)	/	23923.68 ± 3642.16	24715.86 (21288.11; 27289.55)	/	0.023 *
MSS-R	1	2627.54 ± 472.44	2625.82 (2240.99; 2853.09)	0.163	2427.69 ± 859.05	2419.11 (2253.69; 2800.98)	0.190	0.095
MSS-L	1	2550.62 ± 509.45	2592.87 (2166.05; 2686.81)		2318.82 ± 411.52	2266.80 (2128.95; 2609.66)		0.245
MSS-Tot ^a^	1	5178.16 ± 940.73	5198.35 (4359.62; 5754.33)	/	4746.51 ± 1079.35	4814.97 (4324.41; 5346.92)	/	0.173
MSS-R	2	2922.01 ± 704.82	2848.49 (2310.30; 3338.45)	0.513	2796.29 ± 601.49	2653.18 (2372.07; 3326.60)	0.727	0.768
MSS-L	2	2879.33 ± 689.13	2875.83 (2337.30; 3369.11)		2826.91 ± 529.21	2862.92 (2519.62; 3250.57)		0.932
MSS-Tot ^a^	2	5801.34 ± 1159.55	5809.96 (4649.24; 6454.23)	/	5623.2 ± 1021.78	5477.35 (4966.67; 6496.63)	/	0.862
MSS-R	3	4119.71±1077.28	3835.41 (3272.23; 5404.33)	0.464	3571.66 ± 635.21	3756.41 (3658.72; 4342.12)	0.117	0.049 *
MSS-L	3	4184.08 ±1036.80	4053.72 (3197.29; 4714.99)		3615.59 ± 492.46	3929.09 (3193.67; 4046.57)		0.041 *
MSS-Tot ^a^	3	8303.79 ± 1785.91	7369.94 (6623.68; 9866.96)	/	7367.25 ± 1057.09	7708.17 (6870.08; 8273.06)	/	0.043 *
LMW-R	1	27.13 ± 4.64	27.64 (24.12; 30.02)	0.783	26.51 ± 2.88	27.08 (24.10; 29.03)	0.142	0.567
LMW-L	1	27.28 ± 4.93	27.08 (24.00; 31.58)		25.91 ± 2.94	26.74 (25.09; 27.42)		0.289
LMW-R	2	27.89 ± 4.63	28.21 (24.56; 30.56)	0.544	26.82 ± 3.24	26.69 (23.32; 28.43)	0.093	0.107
LMW-L	2	27.51 ± 2.67	28.17 (24.92; 29.49)		26.98 ± 3.21	27.97 (24.13; 29.91)		0.650
LMW-R	3	31.31 ± 3.53	32.30 (28.60; 34.25)	0.807	28.04 ± 2.71	29.32 (28.18; 30.56)	0.174	0.002 *
LMW-L	3	31.19 ± 3.08	31.27 (29.24; 36.96)		27.93 ± 2.49	27.60 (28.29; 30.21)		< 0.001 *
LMD-R	1	34.36 ± 2.90	34.08 (31.59; 36.95)	0.40	32.61 ± 3.74	33.45 (31.05; 35.29)	0.191	0.099
LMD-L	1	33.65 ± 3.39	33.93 (32.22; 36.39)		34.13 ± 6.60	34.09 (31.46; 35.34)		0.987
LMD-R	2	35.67 ± 3.47	35.28 (32.73; 38.57)	0.271	34.68 ± 2.22	34.48 (33.38; 34.63)	0.038	0.097
LMD-L	2	35.32 ± 3.14	34.95 (33.99; 38.26)		33.90 ± 2.77	33.66 (33.59; 35.64)		0.481
LMD-R	3	37.99 ± 4.26	39.19 (35.54; 41.53)	0.037	35.90 ± 2.32	36.12 (35.20; 38.17)	0.672	0.013 *
LMD-L	3	39.48 ± 2.60	40.52 (38.12; 41.92)		36.13 ± 1.92	35.26 (34.16; 38.59)		< 0.001 *
LMH-R	1	28.09 ± 4.32	28.80 (24.01; 30.81)	0.233	26.52 ± 2.59	26.81 (26.00; 28.11)	0.817	0.095
LMH-L	1	27.68 ± 5.25	27.81 (24.00; 31.21)		26.42 ± 2.45	26.82 (24.02; 27.60)		0.232
LMH-R	2	30.15 ± 4.02	29.61 (27.22; 32.41)	0.921	29.45 ± 3.40	29.21 (26.83; 32.23)	0.277	0.609
LMH-L	2	30.11 ± 3.84	30.01 (27.20; 32.40)		29.78 ± 3.51	30.02 (27.82; 32.50)		0.874
LMH-R	3	36.36 ± 3.92	36.40 (33.03; 38.81)	0.900	35.46 ± 3.43	35.21 (34.01; 38.81)	0.103	0.617
LMH-L	3	36.29 ± 4.71	37.72 (32.65; 39.61)		35.98 ± 3.32	36.41 (35.77; 38.02)		0.956

*: Statistically significant difference (*p* < 0.05); Abbreviations: R = right; L = Left; SD = Standard Deviation; ^a^ Sum between right and left maxillary sinus values.

**Table 2:** Comparisons between groups in female subjects.

Measurements	Mean Ranks			Kruskal–Wallis Test	Pairwise Comparisons (Bonferroni Correction)		
	Group 1 6–8 years	Group 2 9–11 years	Group 3 12–14 years	*p* Value	*p* Value Group 1 vs. 2	*p* Value Group 2 vs. 3	*p* Value Group 1 vs. 3
MSV R	21.30	32.91	59.04	<0.001 *	0.060	<0.001 *	<0.001 *
MSV L	20.78	34.91	57.84	<0.001 *	0.066	<0.001 *	<0.001 *
MSV Tot ^a^	20.67	33.73	59.00	<0.001 *	0.034 *	<0.001 *	<0.001 *
MSS R	23.44	32.36	57.20	<0.001 *	0.148	<0.001 *	<0.001 *
MSS L	24.37	33.34	58.10	<0.001 *	0.075	0.002 *	<0.001 *
MSS Tot ^a^	21.89	34.27	57.20	<0.001 *	0.045 *	<0.001 *	<0.001 *
LMW-R	31.44	38.05	46.60	0.001 *	0.821	0.121	0.001 *
LMW-L	30.85	37.36	44.80	0.045 *	1.00	0.210	0.011 *
LMD-R	28.63	34.45	46.40	<0.001 *	0.647	0.091	0.001 *
LMD-L	29.89	35.82	47.20	0.013 *	0.337	0.070	0.004 *
LMH-R	19.96	34.14	59.40	<0.001 *	0.022 *	<0.001 *	<0.001 *
LMH-L	18.91	35.66	59.20	<0.001 *	0.020 *	<0.001 *	<0.001 *

*: Statistically significant difference (*p* < 0.05); Abbreviations: R = right; L = Left; SD = Standard Deviation; ^a^ Sum between right and left maxillary sinus values.

**Table 3:** Comparisons between groups in male subjects.

Measurements	Mean Ranks			Kruskal–Wallis Test	Pairwise Comparisons (Bonferroni Correction)		
	Group 1 6–8 years	Group 2 9–11 years	Group 3 12–14 years	*p* Value	*p* Value Group 1 vs. 2	*p* Value Group 2 vs. 3	*p* Value Group 1 vs. 3
MSV R	26.11	30.65	56.76	<0.001 *	1.000	<0.001 *	<0.001 *
MSV L	25.64	31.35	56.62	<0.001 *	0.998	<0.001 *	<0.001 *
MSV Tot ^a^	25.79	30.74	57.10	<0.001 *	1.000	<0.001 *	<0.001 *
MSS R	24.96	33.43	55.24	<0.001 *	0.451	0.002 *	<0.001 *
MSS L	23.36	34.30	56.43	<0.001 *	0.189	0.001 *	<0.001 *
MSS Tot ^a^	23.64	33.78	56.62	<0.001 *	0.255	0.001 *	<0.001 *
LMW-R	29.18	33.07	50.02	0.002 *	1.000	0.022 *	0.002 *
LMW-L	28.43	34.74	49.19	0.002 *	0.284	0.025 *	0.001 *
LMD-R	24.75	31.96	57.14	<0.001 *	0.221	<0.001 *	<0.001 *
LMD-L	30.57	30.24	51.26	0.001 *	1.000	0.002 *	0.003 *
LMH-R	24.71	32.74	56.33	<0.001 *	0.519	0.001 *	<0.001 *
LMH-L	24.93	33.52	55.19	<0.001 *	0.433	0.002 *	<0.001 *

*: Statistically significant difference *(p* < 0.05); Abbreviations: R = right; L = Left; SD = Standard Deviation; ^a^ Sum between right and left maxillary sinus values.

**Table 4:** Intra-operator and inter-operator agreement for maxillary sinus measurements.

Measurements	ICC (Intra-Operator)	ICC (Inter-Operator)
MSV R	0.981	0.974
MSV L	0.974	0.971
MSV Tot ^a^	0.971	0.982
MSS R	0.982	0.974
MSS L	0.984	0.978
MSS Tot ^a^	0.978	0.983
LMW-R	0.983	0.951
LMW-L	0.967	0.972
LMD-R	0.972	0.965
LMD-L	0.985	0.979
LMH-R	0.989	0.958
LMH-L	0.978	0.944

Abbreviations: R = right; L = Left; ^a^ Sum between right and left maxillary sinus values.
